# Convolutional neural networks for PET functional volume fully automatic segmentation: development and validation in a multi-center setting

**DOI:** 10.1007/s00259-021-05244-z

**Published:** 2021-03-27

**Authors:** Andrei Iantsen, Marta Ferreira, Francois Lucia, Vincent Jaouen, Caroline Reinhold, Pietro Bonaffini, Joanne Alfieri, Ramon Rovira, Ingrid Masson, Philippe Robin, Augustin Mervoyer, Caroline Rousseau, Frédéric Kridelka, Marjolein Decuypere, Pierre Lovinfosse, Olivier Pradier, Roland Hustinx, Ulrike Schick, Dimitris Visvikis, Mathieu Hatt

**Affiliations:** 1 LaTIM, INSERM, UMR 1101, University Brest, Brest, France; 2 GIGA-CRC in vivo Imaging, University of Liège, Liège, Belgium; 3 Department of Radiology, McGill University Health Centre (MUHC), Montreal, Canada; 4 Department of Radiation Oncology, McGill University Health Centre (MUHC), Montreal, Canada; 5 Gynecology Oncology and Laparoscopy Department, Hospital de la Santa Creu i Sant Pau, Barcelona, Spain; 6 Department of Radiation Oncology, Institut de Cancérologie de l’Ouest (ICO), Nantes, France; 7 Nuclear Medicine Department, University Hospital, Brest, France; 8 Nuclear Medicine Department, Institut de Cancérologie de l’Ouest (ICO), Nantes, France; 9 Division of Oncological Gynecology, University Hospital of Liège, Liège, Belgium; 10 Division of Nuclear Medicine and Oncological Imaging, University Hospital of Liège, Liège, Belgium

**Keywords:** Convolutional neural network, PET, Segmentation, Cervical cancer, U-Net

## Abstract

**Purpose:**

In this work, we addressed fully automatic determination of tumor functional uptake from positron emission tomography (PET) images without relying on other image modalities or additional prior constraints, in the context of multicenter images with heterogeneous characteristics.

**Methods:**

In cervical cancer, an additional challenge is the location of the tumor uptake near or even stuck to the bladder. PET datasets of 232 patients from five institutions were exploited. To avoid unreliable manual delineations, the ground truth was generated with a semi-automated approach: a volume containing the tumor and excluding the bladder was first manually determined, then a well-validated, semi-automated approach relying on the Fuzzy locally Adaptive Bayesian (FLAB) algorithm was applied to generate the ground truth. Our model built on the U-Net architecture incorporates residual blocks with concurrent spatial squeeze and excitation modules, as well as learnable non-linear downsampling and upsampling blocks. Experiments relied on cross-validation (four institutions for training and validation, and the fifth for testing).

**Results:**

The model achieved good Dice similarity coefficient (DSC) with little variability across institutions (0.80 ± 0.03), with higher recall (0.90 ± 0.05) than precision (0.75 ± 0.05) and improved results over the standard U-Net (DSC 0.77 ± 0.05, recall 0.87 ± 0.02, precision 0.74 ± 0.08). Both vastly outperformed a fixed threshold at 40% of SUVmax (DSC 0.33 ± 0.15, recall 0.52 ± 0.17, precision 0.30 ± 0.16). In all cases, the model could determine the tumor uptake without including the bladder. Neither shape priors nor anatomical information was required to achieve efficient training.

**Conclusion:**

The proposed method could facilitate the deployment of a fully automated radiomics pipeline in such a challenging multicenter context.

**Supplementary Information:**

The online version contains supplementary material available at 10.1007/s00259-021-05244-z.

## Introduction

Combined positron emission tomography/computed tomography (PET/CT) imaging is widely used in clinical practice to provide functional information on organs and tissues, as well as disease abnormalities. Static PET images provide semi-quantitative information regarding the distribution of a radiotracer uptake. In oncology, fluorodeoxyglucose (FDG) PET imaging is routinely relied upon for diagnosis, staging, treatment planning, and therapy follow-up [20].

In clinical applications, nuclear medicine physicians carry out qualitative assessments of PET/CT images, which is typically sufficient for detecting and anatomically locating lesions. For radiotherapy treatment planning, radiation oncologists manually draw boundaries on fused PET/CT images to determine the gross target volume (GTV) of a tumor, in order to subsequently deliver a specific dose to the target. The boundary of the target should be defined as accurately as possible to maximize the coverage of the target and minimize the dose delivered to surrounding healthy tissues and nearby organs-at-risk (OAR).

More quantitative assessment of FDG uptake in PET images can also be performed. For instance, radiomics analyses [23] aim at extracting clinically relevant measurements through the calculation of numerous image-derived features features (e.g., intensity, shape, and textural). Such measures can subsequently be used to build models predictive of outcome or for assessing changes in tumors before, during, and after treatment in order to better evaluate response to therapy [7, 37, 38]. It has been shown in all image modalities including PET that the choice of the segmentation method in this step of the radiomics workflow can significantly affect the extracted features [21, 31, 42, 46, 51]. In addition, it is recognized that in the absence of fully automated segmentation, this step is a crucial bottleneck and time-consuming step of any radiomics study, preventing such a process to be expanded to very large datasets [23]. There is therefore a need for a delineation method that is not only accurate and robust, but also fully automated as well.

There are several challenges pertaining to PET image segmentation [20]. First, PET images suffer from limited spatial resolution (4–5 mm), comparatively to CT (below 1 mm) due to partial volume effects (PVE) that make boundaries between adjacent functional regions blurred and result in underestimated activity in small objects of interest. Second, signal-to-noise ratio in PET images is inherently low and affected by a vast array of factors, such as scanner sensitivity, temporal resolution, acquisition mode, scan time, quantity and distribution of tracer, applied corrections (e.g., scatter, attenuation, randoms) and reconstruction algorithm type (e.g., resolution recovery, time of flight) and parameters (e.g., number of iterations). All these issues make things challenging in a multi-center context, i.e., when analyzing PET images acquired using different systems, acquisition protocols, and reconstruction settings. Third, the wide variability in shapes and heterogeneity of lesion uptakes might reduce the generalization of segmentation methods to only some specific cases.

An important aspect in medical image segmentation is that the true boundary of the object of interest (ground truth) is impossible to determine without a complete histopathological analysis of an excised tumor, which can typically be performed only in a small number of cases. In PET, even with a very robust protocol, this approach can only provide approximate co-registration between the histopathology slides and the corresponding 3D PET slices [20]. One way to overcome this is the use of a consensus of several manual segmentations by experts as a surrogate of truth [20]. Unfortunately, manual segmentation is typically a labor-intensive, time-consuming task with low reproducibility, due to the high intra- and interobserver variability [19].

There have been a number of algorithms proposed for PET image segmentation, accounting at different degrees for some of the limitations referred to above [20]. For example, thresholding-based methods, the most simple image segmentation techniques, work on the assumption that different tissue types have specific uptake ranges; therefore, segmentation can be done by comparing individual voxel intensities with a set of thresholds. More advanced methods aim at exploiting statistical differences between uptake regions and surrounding tissues. These include different clustering and classification methods trained on a set of features extracted from PET images, as well as atlas-based [39, 45] and generative models such as Gaussian mixture models (GMM) [2] and Fuzzy Locally Adaptive Bayesian (FLAB) model [16]. Numerous other common image segmentation algorithms have been evaluated for this task using PET uptake only [20]. For the vast majority of these published methods, it is usually assumed that the tumor has been previously isolated in a volume of interest (VOI), i.e., the input to the algorithm is not the entire PET image but a sub-volume containing the object of interest, that is usually manually determined after visually detecting the tumor uptake in the whole image. It should be emphasized that numerous approaches tried to improve PET segmentation by considering both PET and CT modalities together, assuming an (almost) perfect correspondence between tumor functional uptake and tumor anatomical boundaries as determined on CT images using co-segmentation approaches exploiting co-registered PET and CT images [9, 14, 34]. This assumption may not be true as radiotracer uptake and anatomical boundaries can be uncorrelated. This also makes the method sensitive to registration issues in PET imaging, especially in body regions affected by motion [20].

Convolutional neural networks (CNNs) have been successfully applied to different medical imaging tasks [35], such as reconstruction [32, 43], denoising [10, 44], segmentaton [13, 40], and classification [6]. Most segmentation studies rely on U-Net [47] that is arguably the most popular network for semantic segmentation, and focus on anatomical modalities such as magnetic resonance imaging (MRI) [8, 40] and CT [41, 48]. The limited number of papers dedicated to PET segmentation usually assume a correspondence between functional and anatomical regions in combined PET/CT or PET/MRI imaging [12, 26, 52–54]. The ground truth is usually obtained through manual delineation performed on multimodal images (e.g., training a network to reproduce delineations performed by radiation oncologist that perform this manually on fused PET/CT images). Guo et al. [13] included PET imaging within a CNN-based multimodal image segmentation framework using PET, MR (T1 and T2), and CT images of a publicly available soft tissue sarcoma dataset of 50 patients. Gross tumor volumes were manually annotated in all four imaging modalities. Different fusion networks were used for feature-, classifier-, and decision-level fusion, demonstrating improved performance at a feature level fusion [13].

Considerably less attention has been dedicated to processing PET images as a stand-alone modality. Moreover, a majority of studies have used only datasets with small cohorts of patients from one or two centers and manual delineation as a surrogate of truth. Under these circumstances, some previously published results might be less generalizable due to high heterogeneity of PET images caused, for instance, by scanner type, reconstruction algorithm, and applied post-processing that vary across centers. Huang et al. [26] applied U-Net with minor modifications for head and neck cancer gross tumor volume segmentation on PET/CT images. Results were obtained for a dataset of 22 patients using manual segmentation as a surrogate of truth. Blanc-Durand et al. [3] evaluated U-Net for glioma segmentation on PET images with the fluoroethyl-tyrosine (FET) tracer. Their dataset contained only 37 patients with manually segmented lesions. Leung et al. [33] used a modified U-Net trained on simulated PET images and fine-tuned using a clinical dataset of 160 patients with manual delineations for lung cancer segmentation. In cervical cancer, Chen et al. [5] proposed to combine a 2D CNN and a post-processing step that relies on prior anatomical information on the tumor roundness and its position relative to the bladder. The choice of the 2D network was dictated by the limited size of the available dataset that contained 1176 slices from 50 patients, and the surrogate of truth was also obtained through manual delineation. Within the scope of the recent MICCAI challenge on automatic PET tumor functional volume delineation, the CNN-based method reached the highest score compared to twelve other approaches, among them some of the current state of the art [22].

In this paper, we focused our experiments on cervical cancer. Approximately 570,000 cases of cervical cancer and 311,000 deaths from the disease occurred in 2018 and this type of cancer was the fourth most common cancer in women worldwide [1]. Recently, predictive models relying on textural features from tumor volumes in PET images were able to identify the subset of patients that will suffer from recurrence after treatment with clinically relevant accuracy [37, 46]. This obviously requires accurate delineation of the tumor volume in the PET images and this is achieved without the use of the associated CT image. Due to the anatomical proximity between the cervix and the bladder that generally has similar FDG uptake in PET scans, conventional techniques (e.g., thresholding, region- and boundary-based methods) provide poor results if applied to the whole image without additional prior knowledge or constraints, which is why a VOI excluding physiological uptake usually needs to be provided as input to the method. For instance, the use of FLAB, as described in the radiomic study above [37], requires an expert to first manually define a VOI containing the tumor but excluding the bladder, in which FLAB is then applied to delineate the tumor. However, this step can be quite labor-intensive and time-consuming, especially when the tumor uptake and bladder are very close to each other, hindering the potential clinical translation of these segmentation tools, and in turn the use of the predictive radiomics-based models. A fully automated segmentation step, without the manual determination of the VOI, is therefore highly desirable in that context.

The purpose of our study was thus to propose a U-Net-based model for the fully automated delineation (i.e., without the need for visual detection of their location and manual determination of a VOI) of 3D functional primary tumor volumes in PET images only, especially in the specific context of cervical cancer where the pathological uptake of interest is located close to a physiological one that should not be included (here, the bladder). A secondary objective was to train the network on a reliable ground truth obtained through accurate and robust PET semi-automated segmentation instead of manual delineation. A final objective was to train and evaluate the performance of our model under standard clinical imaging conditions, considering a multi-center patient cohort without any prior standardization in the data acquisition or image reconstruction processes.

**Fig. 1:**
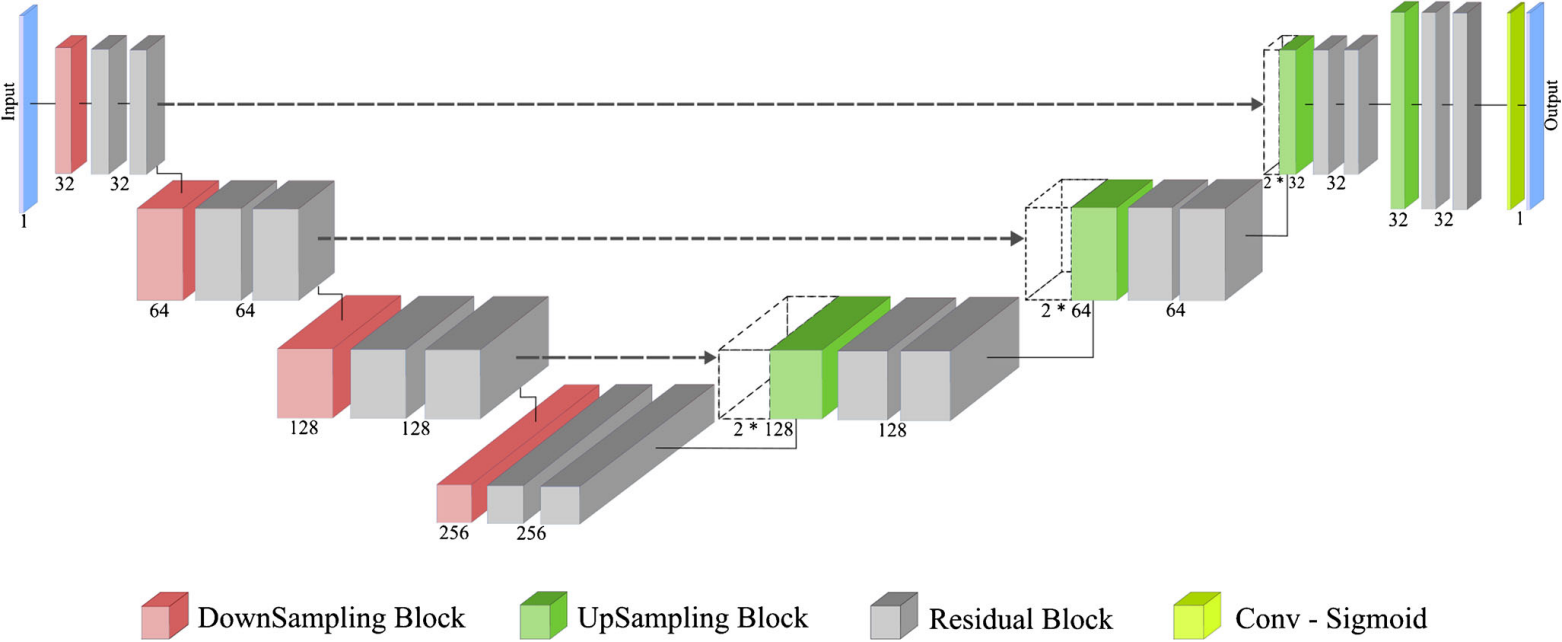
Proposed Encoder-Decoder Network with residual blocks. The number of output channels is depicted under blocks of each group

## Materials and methods

### PET images and training relying on FLAB-derived ground truth

Our first objective is to achieve fully automated determination of the functional uptake boundaries in PET images only, without relying on assumptions regarding its correspondence with anatomical boundaries and to avoid registration issues, which are important in the case of cervical cancer due to the elastic nature of organs in this body region. We decided to train and evaluate the proposed model exclusively on real clinical images, contrary to recent recommendations by the task group 211 of the AAPM (American Association of Physicists in Medicine) dedicated on PET auto-segmentation, which advises to rely on a combination of simulated, phantom, and clinical images [20]. Indeed, usually the number of clinical images available for training and validation is small, and the surrogate of truth is questionable when only manual delineations from experts are available. In such a context, results obtained on large datasets of simulated and/or phantom images can indeed increase the confidence in the results obtained in a smaller amount of clinical cases with less reliable surrogate of truth. However, in the present work, we exploited a large dataset of images that were processed by experts using a semi-automated approach (see section below detailing how the ground truth was generated) for the purpose of radiomics-based outcome modeling studies. In addition, one objective of this work is to evaluate the ability of the proposed approach to deal with fully automated tumor uptake delineation when it is located close to a physiological one that should not be included. Simulated or phantom images corresponding to this specific context are currently not available in large amounts to train and evaluate a CNN-based approach.

We collected a dataset of 232 FDG PET images of patients from five institutions, all with histologically proven cervical cancer, with clinical stage IB1-IVB^1^ (see Supplementary Fig. 1). All images contained the abdominopelvic cavity and were acquired for diagnostic and staging purposes before chemoradiotherapy followed by brachytherapy. Collected images considerably differed in acquisition protocols (scanning duration per bed position, injected radiopharmaceutical dose) and reconstruction (algorithms, use of time of flight information, resolution modeling, voxel and matrix sizes) (see Table 1). Data were corrected for randoms and scatter in all cases. All reconstructed images were corrected for attenuation using the associated low-dose CT.

**Table 1:** Summary of patients, including the different characteristics of the scanners, and associated reconstruction methods and parameters

Institution	Number of patients	Scanner models	Voxel sizes (*mm*^3^)	Reconstruction methods	Time per bed position (s)	FDG total dose (MBq)
University Hospital of Brest, France	69	Siemens Biograph	4.073 × 4.073 × 2.027	PSF+TOF	120 ± 17	253 ± 80
Integrated Centre for Oncology (ICO), France	18 5	Siemens Biograph GE Discovery STE	4.073 × 4.073 × 2.027 4.688 × 4.688 × 3.27	PSF+TOF 3D IR	202 ± 32	210 ± 56
McGill University Health center, Canada	7 19	GE Discovery 710 GE Discovery ST	3.646 × 3.646 × 3.27 5.469 × 5.469 × 3.27	VPFXS OSEM	212 ± 30	398 ± 81
Hospital of the Holy Cross and Saint Paul, Spain	24	Philips Gemini TF	4 × 4 × 4	BLOB-OS-TF	109 ± 21	228 ± 50
University Hospital of Liège, Belgium	90	Philips Gemini TF	4 × 4 × 4	BLOB-OS-TF	73 ± 16	260 ± 32

An objective of our work is to train the network using a reliable ground truth excluding the bladder uptake. Segmentation of the tumor volumes to generate the ground truth was performed on PET images using the FLAB algorithm [16] applied in a semi-automated manner: first, a VOI containing the tumor uptake and excluding the nearby bladder and other physiological uptakes was manually defined by the user. The FLAB algorithm was then run within that VOI to generate a segmentation mask that was reviewed by the user. If this mask was deemed unsatisfactory, the user had the option to re-run the algorithm after specifying different values of initialization parameters in order to obtain a more satisfying result. Finally, the results for all tumors were reviewed and in some cases (< 5%) manually edited before being validated by two experts with more than 15 years of clinical practice. Given that FLAB in such a context has been demonstrated to provide accurate and reliable results in numerous studies [11, 21, 49], including for complex heterogeneous cases [17, 19] and over different scanner model and reconstruction algorithms, especially compared to manual delineation [18], we consider this ground truth sufficiently reliable for the purpose of training and evaluating the proposed approach. Although FLAB was applied only within the manually determined VOI, we then registered the obtained segmentation mask onto the entire PET image used as input to the network for training and testing.

### Network architecture

The widely used 3D U-Net model [4] served as the basis for our network design. Although not the main objective of the present work, we nonetheless introduced three optimizations beyond the standard U-Net model:

1. Original U-Net consists of conventional convolutional blocks composed of a 3 × 3 × 3 convolution, a normalization layer (e.g., batch norm), and a ReLU activation function as a basic element of the network. We chose to rely upon a residual block with full pre-activation [24] supplemented by a concurrent spatial and channel squeeze and excitation (scSE) module (Fig. 1, gray blocks).
2. An important part of the proposed architecture is the integration of squeeze and excitation (SE) blocks that aim at providing the option to compute weights for the feature maps, so the network can put more or less attention on some of them. We implemented SE blocks within the full pre-activation residual blocks, namely a specific modification called concurrent spatial SE (scSE) that has been shown to perform better for image segmentation tasks [48]. In order to include the scSE module in the residual block, we followed the same approach that was applied in SE-ResNet architectures [25]. Due to the high memory consumption working with 3D images, we switched from using batch norm layers to instance normalization (instance norm) that was shown to work better in a small-batch regime [50].
3. We replaced max pooling operations in the encoder of the network by learnable downsampling blocks (Fig. 1, red blocks), which consist of one 3 × 3 × 3 strided convolutional layer, the instance norm, the ReLU activation, and the scSE module. Similarly, we implemented upsampling blocks in the decoder of the network using a 3 × 3 × 3 transposed convolution instead (Fig. 1, green blocks). To reduce memory consumption and increase the receptive field of the network, we implemented the first downsampling block with a kernel size of 7 × 7 × 7 right after the input. The last convolutional layer followed by the sigmoid activation function to produce the model output was applied with a kernel size of 1 × 1 × 1.

## Experimental settings

### Data preprocessing

The PET images exhibited a large variability of voxel sizes (see Table 1) that can adversely affect the model performance since CNNs cannot natively interpret spatial dimensions with different scales. Therefore, we first interpolated all PET images and corresponding segmentation masks to a common resolution of 4 × 4 × 2*mm*^3^ through the use of linear interpolation. A slice thickness of 2 mm was chosen to retain small image details that could be lost if interpolated at a larger voxel size. Linear interpolation was chosen after comparison with other techniques including Nearest Neighbor and B-spline, which led to decreased performance.

PET image intensities can exhibit a high variability in both within image and between images. In order to reduce these variabilities and use them as the input for the CNNs, we applied Z-score normalization for each scan separately, with the mean and the standard deviation computed based only on voxels with non-zero intensities corresponding to the body region.

### Data augmentation

Due to the large variability in shapes, sizes, and heterogeneity of tumor uptakes in PET images, data augmentation can play a useful role in model training. To aid the model learn features invariant to affine transformations that are realistic, we applied mirroring on the axial plane, rotations in random directions with the angle uniformly sampled from the range [5, 15] degrees along the random set of axes, and scaling with a random factor between 0.8 and 1.2. In order to increase the diversity in lesion shapes, we relied on elastic deformations. Gamma correction with *γ* sampled from the uniform distribution between 0.8 and 1.2, and contrast stretching between 0 and 0.8–1.2 of the original range of values was applied to adjust voxel intensity distributions. To improve model robustness, we also added Gaussian noise to training samples. The standard deviation of the noise was equal to 0.1–0.2 standard deviations of the training sample. All augmentation methods were applied independently during model training with a probability of 0.2.

### Training procedure

Due to the large size of PET images, we trained the model on randomly extracted patches of 128 × 128 × 64 voxels with a batch size of 2. Since all PET images corresponded to the abdominopelvic cavity with a number of axial slices ranging from 77 to 192, the chosen patch size was large enough to cover a significant part of the input PET image for all patients.

We trained the model for 400 epochs using Adam optimizer with β_1_ = 0.9 and β_2_ = 0.99 for exponential decay rates for moment estimates. We applied a cosine annealing schedule [36], gradually reducing the learning rate from *lr*_*max*_ = 10^− 4^ to *lr*_*min*_ = 10^− 6^ for every 25 epochs and performing the adjustment at each epoch.

Considering the fact that the Dice similarity coefficient (DSC) is one of the most common metrics used for the evaluation in medical image segmentation, we trained the model with the Soft Dice Loss. Based on [40], in case of binary segmentation, the loss function for one training example can be written as

1 $$ L(y, \hat{y}) = 1 - \frac{2{\sum}_{i}^{\mathcal{N}} y_{i} \hat{y}_{i} + 1}{{\sum}_{i}^{\mathcal{N}} {y_{i}^{2}} + {\sum}_{i}^{\mathcal{N}} \hat{y}_{i}^{2} + 1} $$

where *y*_*i*_ ∈{0,1} is the the binary label for the *i-th* voxel; $\hat {y}_{i} \in [0, 1]$ predicted probability for the *i-th* voxel. Additionally, we applied Laplacian smoothing by adding + 1 to the numerator and the denominator in the loss function to avoid the zero division in cases when only one class is represented in the training example.

### Multi-center cross-validation

Cross-validation is probably the simplest and most widely used method for estimating the expected prediction error of a model on an independent test sample [15]. Importantly, cross-validation is based on the assumption that data samples in the train and test folds are drawn from the same distribution. However, as already mentioned in section “Network architecture,” no standardization in the acquisition or reconstruction protocols were implemented across the five institutions in which the images were collected. In addition, different PET/CT imaging devices with variable overall performance were used in these centers. Therefore, in order to obtain a more reliable estimate of the model performance, we implemented 5-fold cross-validation where each fold was composed only of samples from one of the 5 centers. This simulated a “real-life scenario” in which data from one or several centers are used for training and evaluating a model that is then used in yet another center. For each cross-validation split of the data, we randomly set aside 20% of training samples to tune hyperparameters of the model and to assess the model performance during training.

### Evaluation metrics

Aside from the DSC metric quantifying global volume overlap, we used precision (a.k.a. positive predictive value) *P* and recall *R* (a.k.a. sensitivity) to further evaluate model performance, as recommended by the TG211 [20], where DSC can be written as the harmonic mean of precision and recall:

2 $$ DSC = 2 \frac{P\cdot R}{P+R} $$

Using these metrics, we compared our proposed network to the standard U-Net (StdU-Net) as a baseline model. In addition, a comparison was made with the use of a fixed thresholding method (based on 40% of the maximum standardized uptake within the tumor, denoted from here onwards as T40), still widely used in the literature despite its obvious limitations [20, 22].

## Results and discussion

The results of all methods are summarized in Table 2 and Supplementary Table 1. As expected, T40 obtained poor performance across all test folds compared to stdU-Net and the proposed model. On average, our proposed model outperformed its StdU-Net counterpart in terms of DSC (0.80 vs. 0.77), with a slighlty smaller spread (0.03 vs. 0.05). The largest difference between the proposed method and its standard counterpart was measured on the “Brest” test fold, where StdU-Net demonstrated relatively poorer performance (0.77 vs. 0.68). However, on the other test folds, both models achieved closer results. The superiority of the proposed model was due to a better recall (0.90 vs. 0.87), whereas the difference in terms of precision was smaller (0.75 vs. 0.74). Kolmogorov-Smirnov and Wilcoxon signed-rank tests both indicated that the difference in predictions of two models was significant (*α* = 0.05) only for all evaluation metrics on the Brest test fold, and for recall on “Nantes” (see Supplementary Table 1).

**Table 2:** Segmentation results obtained on the different test folds with the use of cross-validation

Metrics	Model	Test fold					Average
		Brest (n = 69)	Nantes (n = 23)	Montreal (n = 26)	Barcelona (n = 24)	Liège (n = 90)	
DSC	*T*40	0.33 ± 0.36	0.57 ± 0.41	0.37 ± 0.31	0.22 ± 0.24	0.18 ± 0.22	0.33 ± 0.15
	StdU-Net	0.68 ± 0.20	0.79 ± 0.12	0.77 ± 0.13	0.83 ± 0.10	0.79 ± 0.13	0.77 ± 0.05
	Proposed	0.77 ± 0.15	0.81 ± 0.13	0.77 ± 0.21	0.84 ± 0.11	0.79 ± 0.13	0.80 ± 0.03
Precision	*T*40	0.30 ± 0.37	0.56 ± 0.43	0.29 ± 0.28	0.18 ± 0.21	0.14 ± 0.21	0.30 ± 0.16
	StdU-Net	0.61 ± 0.24	0.75 ± 0.15	0.74 ± 0.16	0.81 ± 0.17	0.79 ± 0.18	0.74 ± 0.08
	Proposed	0.69 ± 0.20	0.73 ± 0.16	0.77 ± 0.22	0.81 ± 0.18	0.77 ± 0.19	0.75 ± 0.05
Recall	*T*40	0.48 ± 0.43	0.74 ± 0.35	0.65 ± 0.40	0.38 ± 0.36	0.38 ± 0.37	0.52 ± 0.17
	StdU-Net	0.88 ± 0.15	0.87 ± 0.10	0.85 ± 0.15	0.90 ± 0.09	0.84 ± 0.14	0.87 ± 0.02
	Proposed	0.93 ± 0.10	0.96 ± 0.04	0.83 ± 0.19	0.91 ± 0.08	0.87 ± 0.14	0.90 ± 0.05

The proposed model was compared to the standard U-Net model and the fixed thresholding method in terms of DSC, precision and recall. The mean and standard deviation of each metric on the test folds are computed across corresponding data samples. Average results are reported across the test folds

This finding is in line with previous observations that, when properly tuned, the standard U-Net model can provide highly competitive results in many image segmentation tasks, especially in medical imaging. For example, top-ranked results were obtained in recent segmentation challenges using the ordinary U-Net model [28–30]. Under these circumstances, each step in the entire pipeline (data preprocessing, data augmentation, training procedure, etc.) may have a much larger impact on the model performance than a careful or complex re-design of the model architecture. For instance, we observed in our experiments that applying b-spline interpolation for image resampling instead of linear interpolation deteriorated both models’ performance by an average of nearly 8.5% on the test folds.

**Fig. 2:**
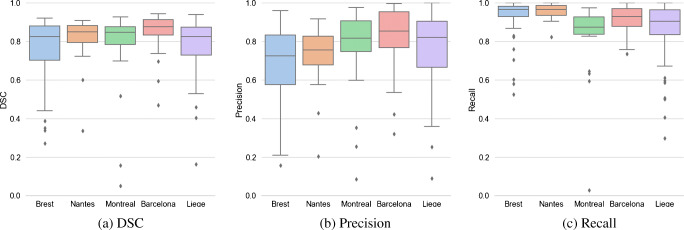
Box plots of the results on the test folds

**Fig. 3:**
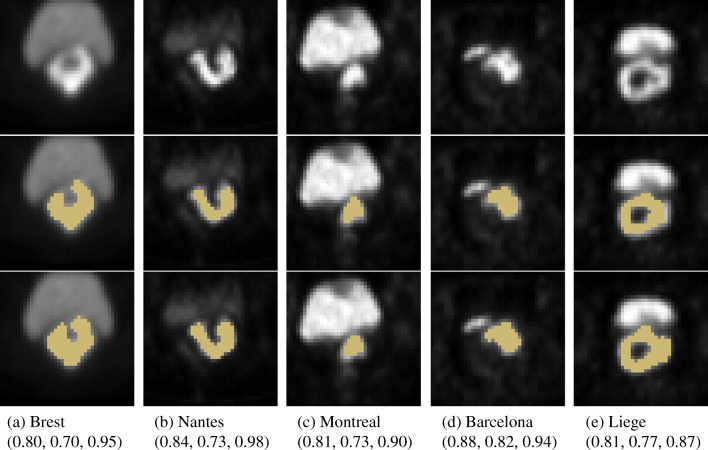
Examples of the model predictions in each test fold. Axial slices. (first row) Input images, (second row) input images with ground truth segmentation, (last row) input images with predicted segmentation. Evaluation metrics for whole scans are provided in format (DSC, precision, recall)

Both models achieved higher recall (between 0.83 and 0.93 on average) than precision (between 0.61 and 0.81) in all test folds, showing a trend in overestimating the ground truth rather than underestimating it. One of the most challenging aspects pertaining to cervical cancer segmentation in PET images is to distinguish the tumor uptake from the adjacent bladder uptake. In all cases, even when the tumor was very close to the tumor, the proposed approach was capable of address this problem independently of the size, location, and shape of the tumor uptake (see examples in Fig. 3). However, the wider spread of results on the two largest test folds (Brest, Liège) (see Fig. 2) could mean that the applied data augmentation techniques are not able to completely mimic all possible variations in presented PET images and alternatives should be investigated, such as, for example, relying on realistic simulated PET images to add more data for training. In addition, due to our 5-fold evaluation scheme based on clinical centers, the size of training sets (and as a result the variety of encountered examples) varied substantially (e.g., holding out Liege yields 146 training cases whereas holding out Nantes yields 209), which could also contribute in explaining these differences (Fig. 3).

**Fig. 4:**
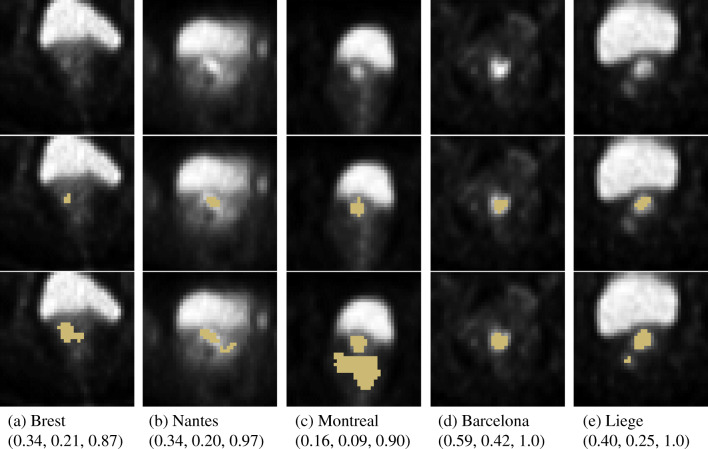
Examples of outliers in each test fold. Axial slices. (first row) Input images, (second row) input images with ground truth segmentation, (last row) input images with predicted segmentation. Evaluation metrics for whole scans are provided in format (DSC, precision, recall)

Analyzing predictions of the models, we identified a number of outliers^2^ in each test fold (see examples in Fig. 4). When considering the DSC metric, the total number of outliers in the entire dataset was equal to 15 (12 for StdU-Net) and varies from 2 to 4 across the test folds. In most cases, the model failed to accurately segment images with relatively small tumor regions (see Fig. 4a, d). More specifically, 11 outliers corresponded to cases where the tumor size was less than 200 voxels (i.e., 6.4 cm^3^), whereas the average value across the dataset was 1160 voxels (i.e., 37.12 cm^3^). The other source of errors in the model predictions is the presence of surrounding tissues with a relatively high uptake that can be misclassified as the tumor (see Fig. 4b, c, e).

The performance was affected by tumor volume (see Supplementary Table 2 and Supplementary Fig. 2): the lowest results were obtained in the first decile group^3^ with DSC = 0.56 compared to the performance obtained on larger tumor volumes (significantly higher between 0.71 and 0.85). This happened due to precision that was steeply increasing along with the tumor size (0.44 to 0.90), whereas recall remained relatively stable (between 0.81 and 0.94). Examining the impact of the tumor contrast^4^, we found that the proposed model demonstrated the worst results on low contrast images (see Supplementary Table 4 and Supplementary Fig. 4). The decile group with the lowest tumor contrast had DSC = 0.67 and recall = 0.77, which were significantly different from the results on other groups (0.77 to 0.84, and 0.88 to 0.93, respectively). Investigating the relation between FIGO stages and the model performance, we did not find significant differences with DSC ranging from 0.75 to 0.82 (see Supplementary Table 4 and Supplementary Fig. 4).

It should be emphasized that although we used a previously well-validated approach to define the ground-truth, this remains a surrogate of truth. In the absence of perfectly registered histopathological spatial information, this is the best we can achieve with a single segmentation method, which obviously provides imperfect results in a small number of cases (for instance highly heterogeneous or very small and low contrast cases) [22]. An even better approach would consist in generating several manual delineations by experts (at least three) in addition to the results of FLAB (other algorithms with proven good performance [22] could be added too) and generating a statistical consensus of all these segmentation results. This would provide the proposed model an even more reliable ground truth to learn from, but it would be considerably more time-consuming and tedious, especially for generating the numerous manual delineations. Alternatively, our approach consisting in training the network on rigorously determined ground-truth masks could be reproduced by relying on other semi-automated methods with similar demonstrated levels of performance [20, 22]. Once trained, the proposed network can be applied to new data instantaneously, without the need for user intervention beyond checking and validating the output result.

Unlike [5], we did not rely on any post-processing techniques built on prior anatomical information. First, based on the segmentation results of the proposed model, it appears able to natively learn the anatomic position of the tumor relative to the bladder from training samples without additional a priori guidance. Second, the assumption about the tumor roundness contradicts numerous examples in our dataset, especially those with heterogeneous distributions (see Fig. 3b, e). Although in the present case we focused on PET-only delineation, the proposed model can be trained using multiple different modalities as input. It might be beneficial in specific cases, such as dealing with small and/or low-contrast tumors, to extract additional information from associated CT or MRI modalities. For instance, in the context of the MICCAI 2020 Head and Neck Tumor segmentation challenge (HECKTOR), we applied a similar U-Net-based model to delineate lesions in combined PET/CT images, reaching 1st rank performance with DSC of 0.76 [27]. However, the main challenge in that case is to have a reliable ground truth determined on fused multimodal data, which could prove quite difficult in the cervical region due to anatomical deformations and differences between PET and CT datasets.^5^

With respect to our original objectives, our results obtained with the use of multi-center cross-validation allow concluding that the designed model is able to provide similar performance on PET images from different institutions and is robust to variations in scanner types, reconstruction algorithms, and post-processing methods. In addition, it allows for fully automated delineation of the tumor uptake without the need to exclude the bladder uptake, either manually or through the incorporation of additional prior information or constraints.

## Conclusion

In this work, we trained a modified U-Net model for fully automatic tumor uptake delineation in PET images in a multi-center context, without the need for additional anatomical information or prior constraints. The ability of the proposed model to learn and perform well for this task was demonstrated in PET images of 232 patients collected from five institutions. The ground-truth labels for all patients were generated by experts with the use of a semi-automated algorithm, to reduce observer-related variability and to avoid relying on manual delineations. We presented a versatile pipeline that includes appropriate data preprocessing and augmentation, design of the model architecture beyond the standard U-Net model, and an optimized training procedure. We mimicked a typical clinical scenario and conducted all experiments in a multi-center context. The designed model obtained good average accuracy for all considered institutions with very small standard deviation (DSC of 0.80 ± 0.03) without requiring any change in the pipeline. It slightly improved accuracy over the standard U-Net model, although both approaches provided good results and largely outperformed the fixed threshold approach. The described approach managed to avoid including the bladder uptake in the resulting segmentation without the need for additional anatomical information (for instance using the CT image) or priors such as shape constraints, and can therefore achieve fully automated delineation of the tumor uptake without the need for any user intervention. It can be implemented with minimal modifications to solve a variety of other segmentation tasks in different medical imaging modalities and could facilitate the deployment of fully automated radiomics pipelines.

## Supplementary Information

ESM 1 (PDF 894 KB)

## Footnotes

1 According to the staging system of the International Federation of Gynecology and Obstetrics (FIGO).

2 A data point *x*_*i*_ from a dataset $X = \{x_{1}, \dots , x_{n}\}$ is an *outlier*, if *x*_*i*_ < *q*_1_ + 1.5(*q*_3_ − *q*_1_), where *q*_*i*_ is the *i*-th empirical quartile of X.

3 The first decile group corresponds to 10% of patients with the smallest tumors.

4 We roughly estimated tumor contrast as the ratio between average tumor intensity and average intensity of the body region.

5 In the HECKTOR challenge, manual delineation performed by a single expert was used as the ground truth.

## References

[1] Arbyn M, Weiderpass E, Bruni L, de Sanjosé S, Saraiya M, Ferlay J, Bray F (2020). Estimates of incidence and mortality of cervical cancer in 2018: a worldwide analysis. Lancet Glob Health.

[2] Aristophanous M, Penney BC, Martel MK, Pelizzari CA (2007). A gaussian mixture model for definition of lung tumor volumes in positron emission tomography. Med Phys.

[3] Blanc-Durand P, Van Der Gucht A, Schaefer N, Itti E, Prior JO. 2018. Automatic lesion detection and segmentation of 18F-FET PET in gliomas: A full 3D U-Net convolutional neural network study. PLoS One. 10.1371/journal.pone.0195798 PMC5898737 29652908

[4] Çiçek Ö, Abdulkadir A, Lienkamp SS, Brox T, Ronneberger O. 2016. 3D U-Net: Learning dense volumetric segmentation from sparse annotation. In: International Conference on Medical Image Computing and Computer-Assisted Intervention, pp 424–432.

[5] Chen L, Shen C, Zhou Z, Maquilan G, Albuquerque K, Folkert MR, Wang J. 2019. Automatic PET cervical tumor segmentation by combining deep learning and anatomic prior. Phys Med Biol. 10.1088/1361-6560/ab0b64 PMC7098064 30818303

[6] Coudray N, Ocampo PS, Sakellaropoulos T, Narula N, Snuderl M, Fenyö D, Moreira AL, Razavian N, Tsirigos A (2018). Classification and mutation prediction from non-small cell lung cancer histopathology images using deep learning. Nat Med.

[7] Desseroit M, Visvikis D, Tixier F, Majdoub M, Perdrisot R, Guillevin R, Cheze Le Rest C, Hatt M (2016). Development of a nomogram combining clinical staging with (18)f-FDG PET/CT image features in non-small-cell lung cancer stage i-III. Eur J Nucl Med Mol Imaging.

[8] Dolz J, Gopinath K, Yuan J, Lombaert H, Desrosiers C, Ben Ayed I. 2018. HyperDense-Net: a hyper-densely connected CNN for multi-modal image segmentation. IEEE Transactions on Medical Imaging. 10.1109/TMI.2018.2878669 30387726

[9] El Naqa I, Yang D, Apte A, Khullar D, Mutic S, Zheng J, Bradley JD, Grigsby P, Deasy J (2007). Concurrent multimodality image segmentation by active contours for radiotherapy treatment planning. Med Phys.

[10] Gong K, Guan J, Liu CC, Qi J (2019). PET image denoising using a deep neural network through fine tuning. IEEE Transactions on Radiation and Plasma Medical Sciences.

[11] Grootjans W, Usmanij EA, Oyen WJ, van der Heijden EH, Visser EP, Visvikis D, Hatt M, Bussink J, de Geus-Oei L (2016). Performance of automatic image segmentation algorithms for calculating total lesion glycolysis for early response monitoring in non-small cell lung cancer patients during concomitant chemoradiotherapy. Radiother Oncol.

[12] Guo Z, Guo N, Gong K, Zhong S, Li Q. 2019. Gross tumor volume segmentation for head and neck cancer radiotherapy using deep dense multi-modality network. Phys Med Biol 64(20). 10.1088/1361-6560/ab440d PMC7186044 31514173

[13] Guo Z, Li X, Huang H, Guo N, Li Q (2019). Deep learning-based image segmentation on multimodal medical imaging. IEEE Transactions on Radiation and Plasma Medical Sciences.

[14] Hanzouli-Ben Salah H, Lapuyade-Lahorgue J, Bert J, Benoit D, Lambin P, Van Baardwijk A, Monfrini E, Pieczynski W, Visvikis D, Hatt M (2017). A framework based on hidden Markov trees for multimodal PET/CT image co-segmentation. Med Phys.

[15] Hastie T, Tibshirani R, Friedman J. 2009. The elements of statistical learning: Data Mining, Inference, and Prediction. Springer Science & Business Media.

[16] Hatt M, Cheze le Rest C, Turzo A, Roux C, Visvikis D (2009). A fuzzy locally adaptive Bayesian segmentation approach for volume determination in PET. IEEE Trans Med Imaging.

[17] Hatt M, Cheze le Rest C, Descourt P, Dekker A, De Ruysscher D, Oellers M, Lambin P, Pradier O, Visvikis D (2010). Accurate automatic delineation of heterogeneous functional volumes in positron emission tomography for oncology applications. Int J Radiat Oncol Biol Phys.

[18] Hatt M, Cheze-le Rest C, Albarghach N, Pradier O, Visvikis D (2011). PET functional volume delineation: a robustness and repeatability study. Eur J Nucl Med Mol Imaging.

[19] Hatt M, Cheze-le Rest C, van Baardwijk A, Lambin P, Pradier O, Visvikis D (2011). Impact of tumor size and tracer uptake heterogeneity in (18)F-FDG PET and CT non-small-cell lung cancer tumor delineation. J Nucl Med.

[20] Hatt M, Lee JA, Schmidtlein C, Naqa I, Caldwell C, De Bernardi E, Lu W, Das S, Geets X, Gregoire V, Jeraj R, MacManus M, Mawlawi O, Nestle U, Pugachev A, Schöder H, Shepherd T, Spezi E, Visvikis D, Zaidi H, Kirov A (2017). Classification and evaluation strategies of auto-segmentation approaches for PET: Report of AAPM task group no. 211. Med Phys.

[21] Hatt M, Laurent B, Fayad H, Jaouen V, Visvikis D, Le Rest CC (2018). Tumour functional sphericity from PET images: prognostic value in NSCLC and impact of delineation method. Eur J Nucl Med Mol Imaging.

[22] Hatt M, Laurent B, Ouahabi A, Fayad H, Tan S, Li L, Lu W, Jaouen V, Tauber C, Czakon J, Drapejkowski F, Dyrka W, Camarasu-Pop S, Cervenansky F, Girard P, Glatard T, Kain M, Yao Y, Barillot C, Visvikis D (2018). The first MICCAI challenge on PET tumor segmentation. Med Image Anal.

[23] Hatt M, Le Rest C, Tixier F, Badic B, Schick U, Visvikis D (2019). Radiomics: d are also images. J Nucl Med.

[24] He K, Zhang X, Ren S, Sun J. 2016. Identity mappings in deep residual networks. In: European conference on Computer Vision (ECCV).

[25] Hu J, Shen L, Albanie S, Sun G, Wu E. 2017. Squeeze-and-excitation networks. arXiv preprint arXiv:170901507. 10.1109/TPAMI.2019.2913372 31034408

[26] Huang B, Chen Z, Wu PW, Ye Y, Feng ST, Wong CYO, Zheng L, Liu Y, Wang T, Li Q, Huang B. 2018. Fully automated delineation of gross tumor volume for head and neck cancer on PET-CT using deep learning. A dual-center study. Contrast Media & Molecular Imaging. 10.1155/2018/8923028 PMC6220410 30473644

[27] Iantsen A, Visvikis D, Hatt M. Squeeze-and-excitation normalization for automated delineation of head and neck primary tumors in combined PET and CT images. HECKTOR 2020. In: Andrearczyk V, Oreiller V, and Depeursinge A, editors. Switzerland: Springer Nature; 2021. p. 1–7.

[28] Isensee F, Maier-Hein KH. 2019. An attempt at beating the 3D U-Net. arXiv preprint arXiv:190802182.

[29] Isensee F, Kickingereder P, Wick W, Bendszus M, Maier-Hein KH. 2018. No New-Net. arXiv preprint arXiv:180910483.

[30] Isensee F, Jaeger PF, Kohl S, Petersen J, Maier-Hein KH. 2020. nnU-Net: a self-configuring method for deep learning-based biomedical image segmentation. Nat Methods. 10.1038/s41592-020-01008-z 33288961

[31] Kocak B, Ates E, Durmaz ES, Ulusan MB, Kilickesmez O (2019). Influence of segmentation margin on machine learning-based high-dimensional quantitative CT texture analysis: a reproducibility study on renal clear cell carcinomas. Eur Radiol.

[32] Lee H, Lee J, Kim H, Cho B, Cho S (2019). Deep-neural-network-based sinogram synthesis for sparse-view CT image reconstruction. IEEE Transactions on Radiation and Plasma Medical Sciences.

[33] Leung KH, Marashdeh W, Wray R, Ashrafinia S, Pomper MG, Rahmim A, Jha AK. 2020. A physics-guided modular deep-learning based automated framework for tumor segmentation in PET. arXiv preprint arXiv:14090473. 10.1088/1361-6560/ab8535 PMC12243949 32235059

[34] Li L, Lu W, Tan Y, Tan S (2019). Variational PET/CT tumor co-segmentation integrated with PET restoration. IEEE Transactions on Radiation and Plasma Medical Sciences.

[35] Litjens G, Tand Bejnordi KBE, Setio A, Ciompi F, Ghafoorian M, van der Laak J, van Ginneken B, Sánchez C (2017). A survey on deep learning in medical image analysis. Med Image Anal.

[36] Loshchilov I, Hutter F. 2016. SGDR: Stochastic gradient descent with warm restarts. arXiv preprint arXiv:160803983.

[37] Lucia F, Visvikis D, Vallières M, Desseroit M, Miranda O, Robin P, Bonaffini PA, Alfieri J, Masson I, Mervoyer A, Reinhold C, Pradier O, Hatt M, Schick U (2019). External validation of a combined PET and MRI radiomics model for prediction of recurrence in cervical cancer patients treated with chemoradiotherapy. Eur J Nucl Med Mol Imaging.

[38] Majdoub M, Hoeben BAW, Troost EGC, Oyen WJG, Kaanders JHAM, Cheze Le Rest C, Visser EP, Visvikis D, Hatt M (2018). Prognostic value of head and neck tumor proliferative sphericity from 3’-deoxy-3’-[18F] fluorothymidine positron emission tomography. IEEE Transactions on Radiation and Plasma Medical Sciences.

[39] Martins S, Bragantini J, Falcão A, Yasuda C (2019). Atlas-based multiorgan segmentation for dynamic abdominal PET. Med Phys.

[40] Milletari F, Navab N, Ahmadi SA. 2016. V-Net: fully convolutional neural networks for volumetric medical image segmentation. In: Fourth International conference on 3D Vision (3DV), pp 565–571 .

[41] Oktay O, Schlemper J, Le Folgoc L, Lee M, Heinrich M, Misawa K, Mori K, McDonagh S, Hammerla NY, Kainz B, Glocker B, Rueckert D. 2018. Attention U-Net: learning where to look for the pancreas. arXiv preprint arXiv:180403999.

[42] Pfaehler E, Beukinga R, de Jong JR, Slart RJA, Slump CH, Dierckx RA, Boellaard R (2019). Repeatability of 18 f-FDG PET radiomic features: a phantom study to explore sensitivity to image reconstruction settings, noise, and delineation method. Med Phys.

[43] Qin C, Schlemper J, Caballero J, Price AN, Hajnal JV, Rueckert D (2019). Convolutional recurrent neural networks for dynamic MR image reconstruction. IEEE Trans Med Imaging.

[44] Ramon AJ, Yang Y, Pretorius PH, Johnson KL, King MA, N WM (2020). Improving diagnostic accuracy in low-dose SPECT myocardial perfusion imaging with convolutional denoising networks. IEEE Trans Med Imaging.

[45] Ren S, Laub P, Lu Y, Naganawa M, Carson RE (2019). Atlas-based multiorgan segmentation for dynamic abdominal PET. IEEE Transactions on Radiation and Plasma Medical Sciences.

[46] Reuzé S, Orlhac F, Chargari C, Nioche C, Limkin E, Riet F, Escande A, Haie-Meder C, Dercle L, Gouy S, Buvat I, Deutsch E, Robert C (2017). Prediction of cervical cancer recurrence using textural features extracted from 18f-FDG PET images acquired with different scanners. Oncotarget.

[47] Ronneberger O, Fischer P, Brox T. 2015. U-Net: convolutional networks for biomedical image segmentation. In: International conference on medical image computing and computer-assisted intervention, pp 234–241.

[48] Roy AG, Navab N, Wachinger C. 2018. Concurrent spatial and channel ‘squeeze & excitation’ in fully convolutional networks. In: International conference on medical image computing and computer-assisted intervention, pp 421–429.

[49] Supiot S, Rousseau C, Dore M, Cheze-Le-Rest C, Kandel-Aznar C, Potiron V, Guerif S, Paris F, Ferrer L, Campion L, Meingan P, Delpon G, Hatt M, Visvikis D (2018). Evaluation of tumor hypoxia prior to radiotherapy in intermediate-risk prostate cancer using 18F-fluoromisonidazole PET/CT: a pilot study. Oncotarget.

[50] Wu Y, He K. 2018. Group normalization. arXiv preprint arXiv:180308494.

[51] Zhang X, Zhong L, Zhang B, Zhang L, Du H, Lu L, Zhang S, Yang W, Feng Q. 2019. The effects of volume of interest delineation on MRI-based radiomics analysis: evaluation with two disease groups. Cancer Imaging 19(1). 10.1186/s40644-019-0276-7 PMC6925418 31864421

[52] Zhao L, Lu Z, Jiang J, Zhou Y, Wu Y, Feng Q (2019). Automatic nasopharyngeal carcinoma segmentation using fully convolutional networks with auxiliary paths on dual-modality PET-CT images. J Digit Imaging.

[53] Zhao X, Li L, Lu W, Tan S (2018). Tumor co-segmentation in PET/CT using multi-modality fully convolutional neural network. Phys Med Biol.

[54] Zhong Z, Kim Y, Zhou L, Plichta K, Allen B, Buatti J, Wu X (2018). 3D fully convolutional networks for co-segmentation of tumors on PET-CT images. Proc IEEE Int Symp Biomed Imaging.

