# Supplementary material for: Convolutional neural networks for PET functional volume fully automatic segmentation: development and validation in a multi-center setting
Source: Eur J Nucl Med Mol Imaging. 2021 Mar 27;48(11):3444–56. doi: 10.1007/s00259-021-05244-z (PMC8440243; doi:10.1007/s00259-021-05244-z)
Supplement: Supplementary file 1 — (PDF 894 KB) [file 259_2021_5244_MOESM1_ESM.pdf]

Supplementary Information

Convolutional neural networks for PET functional volume fully automatic segmentation: development and validation in a multi-center setting

Andrei Iantsen*[1], Marta Ferreira[2], Francois Lucia[1, 3], Vincent Jaouen[1], Caroline Reinhold[5], Pietro Bonaffini[5], Joanne Alfieri[6], Ramon Rovira[7], Ingrid Masson[8], Philippe Robin[4], Augustin Mervoyer[8], Caroline Rousseau[9], Frédéric Kridelka[11], Marjolein Decuypere[11], Pierre Lovinfosse[10], Olivier Pradier[1, 3], Roland Hustinx[2], Ulrike Schick[1, 3], Dimitris Visvikis[1], and Mathieu Hatt†[1]

[1] *LaTIM, INSERM, UMR 1101, University Brest, Brest, France*
[2] *GIGA-CRC in vivo Imaging, University of Liège, Liège, Belgium*
[3] *Radiation Oncology Department, University Hospital, Brest, France*
[4] *Nuclear Medicine Department, University Hospital, Brest, France*
[5] *Department of Radiology, McGill University Health Centre (MUHC), Montreal, Canada*
[6] *Department of Radiation Oncology, McGill University Health Centre (MUHC), Montreal, Canada*
[7] *Gynecology Oncology and Laparoscopy Department, Hospital de la Santa Creu i Sant Pau, Barcelona, Spain*
[8] *Department of Radiation Oncology, Institut de Cancérologie de l'Ouest (ICO), Nantes, France*
[9] *Nuclear medicine department, Institut de Cancérologie de l'Ouest (ICO), Nantes, France*
[10] *Division of Nuclear Medicine and Oncological Imaging, University Hospital of Liège, Liège, Belgium*
[11] *Division of Oncological Gynecology, University Hospital of Liège, Liège, Belgium*

*Corresponding author, email: andrei.iantsen@inserm.fr

†D. Visvikis and M. Hatt contributed equally to this work.

Supplementary Table 1: Kolmogorov-Smirnov and Wilcoxon signed-rank tests to compare results of the proposed model and StdU-Net. Both tests are two-sided and applied to each evaluation metric. Test statistics (T) and corresponding P-values (P) are present in columns. Asterisks indicate statistically significant results with the significance level $\alpha = 0.05$.

Center	Kolmogorov-Smirnov Test						Wilcoxon Signed-Rank Test					
	DSC		Precision		Recall		DSC		Precision		Recall	
	T	P	T	P	T	P	T	P	T	P	T	P
Brest	0.26	.02*	0.25	.03*	0.28	.01*	499	$<$ .001*	716	$<$ .001*	294	$<$ .001*
Nantes	0.30	.24	0.17	.89	0.52	$<$ .001*	74	.05	97	.21	11	$<$ .001*
Montreal	0.23	.50	0.27	.31	0.27	.31	143	.41	103	.07	116	.13
Barcelona	0.29	.26	0.17	.90	0.25	.45	75	.03*	129	.55	88	.08
Liège	0.07	.99	0.11	.64	0.17	.16	1932	.64	1696	.16	900	$<$ .001*

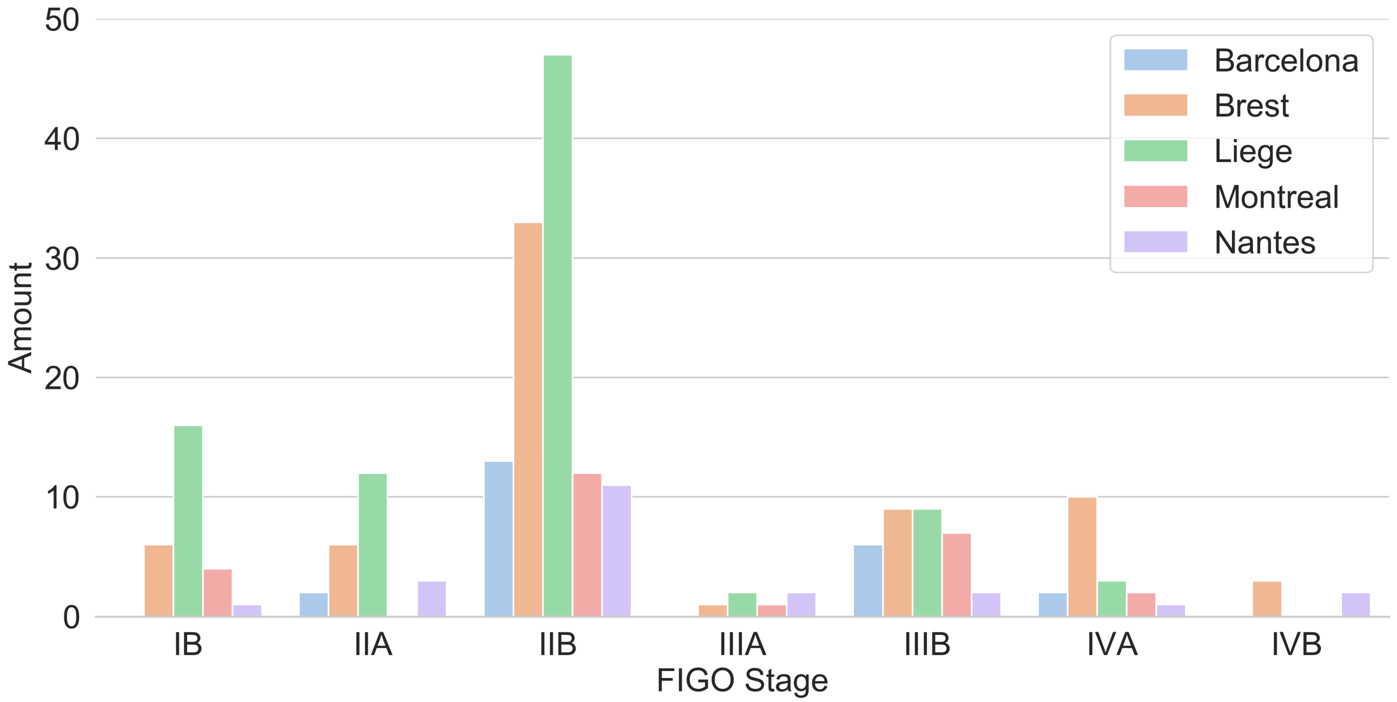

Supplementary Figure 1: Distributions of the patients with different FIGO stages in each center.

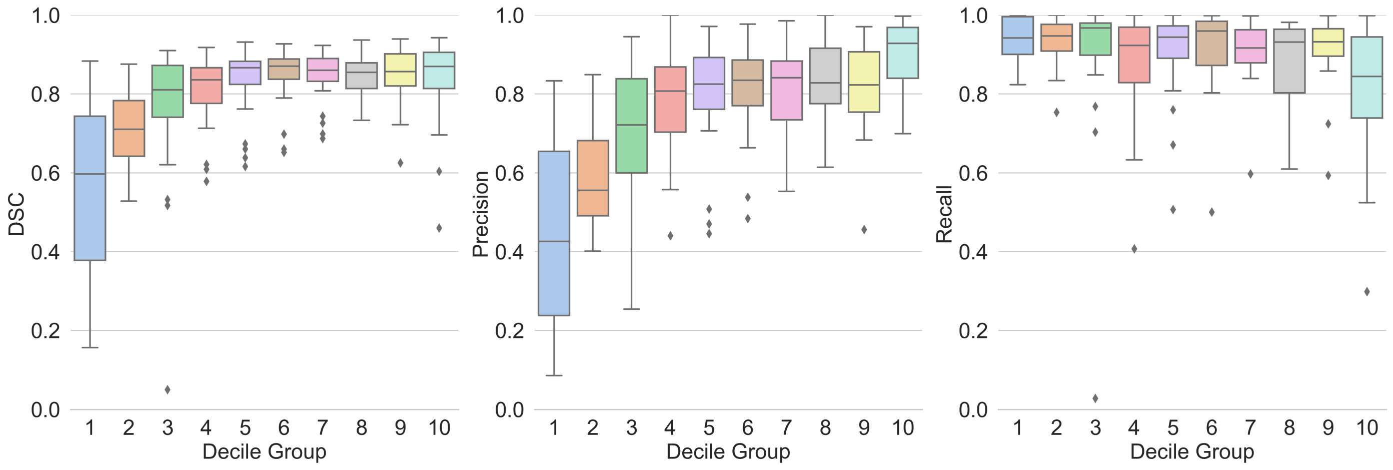

(a) DSC (b) Precision (c) Recall

Supplementary Figure 2: Results of the proposed model for different *volume* decile groups. The *i-th* decile group corresponds to patients with the tumor *volume* between d_{i-1} and d_i, where d_i - the *i-th* empirical decile of the tumor *volume* distribution.

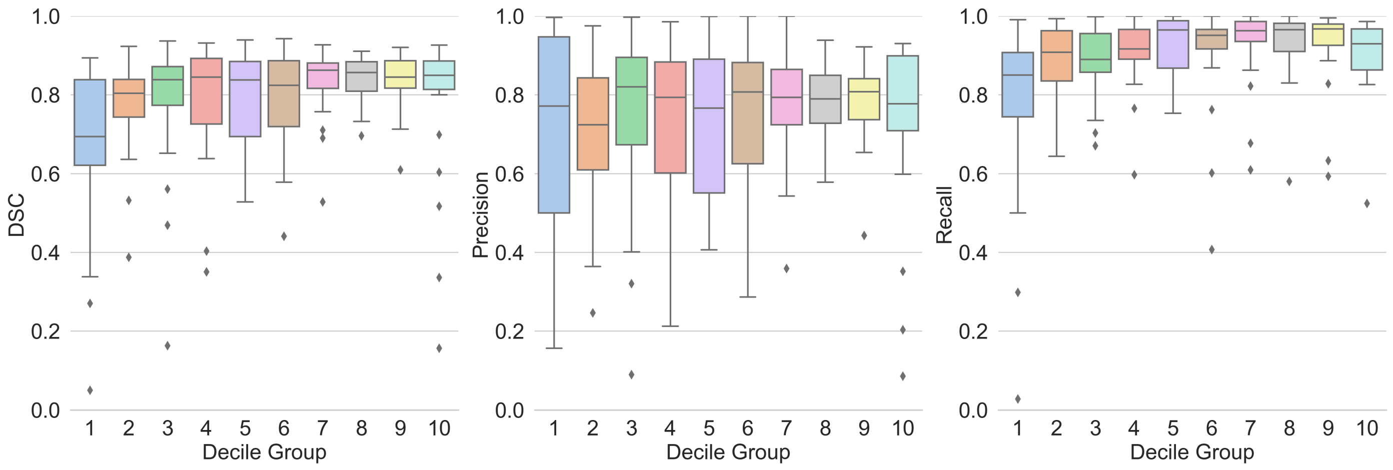

(a) DSC (b) Precision (c) Recall

Supplementary Figure 3: Results of the proposed model for different *contrast* decile groups. The *i-th* decile group corresponds to patients with the tumor *contrast* between d_{i-1} and d_i, where d_i - the *i-th* empirical decile of the tumor *contrast* distribution. The tumor contrast is defined as a ratio of the average tumor intensity to the average intensity of the body region.

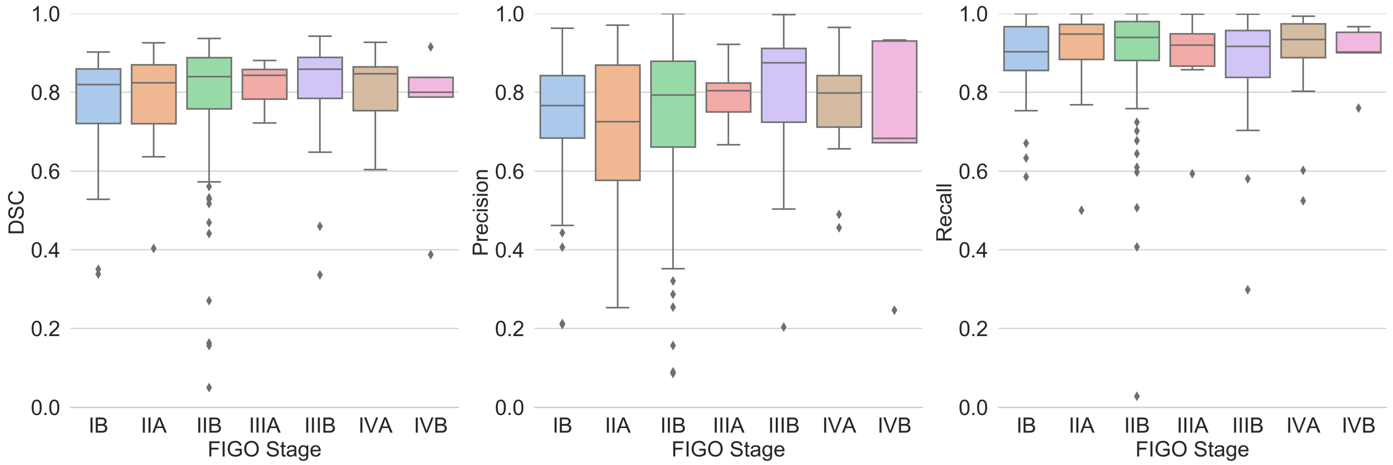

(a) DSC (b) Precision (c) Recall

Supplementary Figure 4: Results of the proposed model for different FIGO stages.

Supplementary Table 2: Average results of the proposed model for different *volume* decile groups. The *i-th* decile group corresponds to patients with the tumor *volume* between d_{i-1} and d_i, where d_i - the *i-th* empirical decile of the tumor *volume* distribution.

Metric	Volume Decile Group									
	1	2	3	4	5	6	7	8	9	10
DSC	0.56 ± 0.22	0.71 ± 0.1	0.76 ± 0.19	0.81 ± 0.1	0.83 ± 0.09	0.84 ± 0.08	0.85 ± 0.07	0.84 ± 0.05	0.85 ± 0.07	0.83 ± 0.12
Precision	0.44 ± 0.23	0.59 ± 0.13	0.69 ± 0.19	0.78 ± 0.14	0.80 ± 0.15	0.81 ± 0.13	0.81 ± 0.12	0.83 ± 0.11	0.82 ± 0.12	0.90 ± 0.10
Recall	0.94 ± 0.06	0.93 ± 0.06	0.89 ± 0.2	0.87 ± 0.14	0.91 ± 0.12	0.91 ± 0.11	0.91 ± 0.08	0.88 ± 0.11	0.91 ± 0.09	0.81 ± 0.18

Supplementary Table 3: Average results of the proposed model for different *contrast* decile groups. The *i-th* decile group corresponds to patients with the tumor *contrast* between d_{i-1} and d_i, where d_i - the *i-th* empirical decile of the tumor *contrast* distribution. The tumor contrast is defined as a ratio of the average tumor intensity to the average intensity of the body region.

Metric	Contrast Decile Group									
	1	2	3	4	5	6	7	8	9	10
DSC	0.67 ± 0.21	0.77 ± 0.12	0.78 ± 0.18	0.78 ± 0.15	0.79 ± 0.13	0.79 ± 0.13	0.83 ± 0.09	0.84 ± 0.06	0.84 ± 0.07	0.78 ± 0.19
Precision	0.70 ± 0.27	0.71 ± 0.20	0.75 ± 0.24	0.73 ± 0.22	0.73 ± 0.20	0.75 ± 0.20	0.78 ± 0.14	0.78 ± 0.09	0.78 ± 0.10	0.74 ± 0.23
Recall	0.77 ± 0.23	0.89 ± 0.09	0.88 ± 0.09	0.91 ± 0.09	0.92 ± 0.09	0.90 ± 0.14	0.93 ± 0.10	0.93 ± 0.09	0.92 ± 0.11	0.90 ± 0.10

Supplementary Table 4: Average results of the proposed model for different FIGO stages.

Metric	FIGO Stage						
	IB (n=27)	IIA (n=23)	IIB (n=120)	IIIA (n=6)	IIIB (n=33)	IVA (n=18)	IVB (n=5)
DSC	0.76 ± 0.15	0.78 ± 0.12	0.79 ± 0.16	0.82 ± 0.06	0.81 ± 0.14	0.81 ± 0.10	0.75 ± 0.21
Precision	0.71 ± 0.20	0.72 ± 0.19	0.74 ± 0.21	0.79 ± 0.09	0.80 ± 0.18	0.77 ± 0.14	0.69 ± 0.28
Recall	0.88 ± 0.11	0.91 ± 0.11	0.90 ± 0.13	0.87 ± 0.15	0.87 ± 0.14	0.89 ± 0.13	0.90 ± 0.08
